# Association between density of convenience and small grocery stores with diet quality in adults living in Mexico City: A cross sectional study

**DOI:** 10.3389/fpubh.2022.857754

**Published:** 2022-08-05

**Authors:** Ana Isabel Rodríguez-Guerra, Nancy López-Olmedo, Catalina Medina, César Hernández-Alcaraz, Ana G. Ortega-Avila, Simón Barquera

**Affiliations:** ^1^Center for Health and Nutrition Research, National Institute of Public Health, Cuernavaca, Mexico; ^2^Center for Population and Health Research, National Institute of Public Health, Cuernavaca, Mexico; ^3^Institute of Geography, National Autonomous University of Mexico, Ciudad de Mexico, Mexico

**Keywords:** food environment, grocery store, convenience store, diet quality, Mexico

## Abstract

There is evidence of the association between different retail stores and food consumption, yet research is still limited in low- and medium-income countries, where the context of the food retail environment is different from that observed in high-income countries. Specifically, less is known about how convenience and small grocery stores, which offer products with immediate access, are associated with the diet as a whole. The present study assessed the association between density of convenience and small grocery stores and diet quality in adults from the Mexico City Representative Diabetes Survey 2015. A final sample size of 1,023 adults aged 20–69 years was analyzed. The density of stores was measured using Euclidean buffers within 500 meters of each participant's home. The Mexican Alternate Healthy Eating Index (MxAHEI) was used to assess diet quality. Multivariable Poisson models were used to test the association of convenience and small grocery stores densities with the MxAHEI. Although our results were not statistically significant, we observed a lower diet quality score among adults from Mexico City living in areas with a higher density of small grocery and convenience stores. More research is needed on the influence of environmental food retail on food consumption.

## Introduction

Low-quality diets have been associated with obesity, cancer, and other cardiometabolic outcomes (1–3). In 2017, low-quality diets accounted for 16% of disability-adjusted life years and were responsible for 22% of global deaths, with cardiovascular disease as the leading cause (3). The global shift toward unhealthy dietary patterns (diets high in saturated and trans-fat, added sugars or sodium, and low in fiber) has been a public health concern. This transition has been accompanied by increased marketing of ultra-processed foods and exponential growth of food retailers (4). The food retail environment, defined as places where people shop for food, is one of the key determinants of diet as it can facilitate the intake of healthy or unhealthy foods (5).

There is evidence of the association between different food retailers and food consumption. In high-income countries (HICs), primary access to supermarkets and limited access to convenience stores-small store with mostly ultra-processed and ready-to-eat foods-has been associated with a healthier diet (6). Convenience stores have been linked with low intake of fruits and whole grains, low diet quality scores in adults, and higher intake of sugar-sweetened beverages in children and adolescents (7–10). However, evidence of the association between food retail environment and food intake could be limited in medium- and low-income countries (LMICs), mainly due to the different contexts (11). For example, within the Latin American context, including Mexico, the food environment is characterized by a high proportion of small retailers. These retailers are mainly family-owned businesses located near homes that regularly offer staple foods, fresh products, as well as a low proportion of ultra-processed products (12–15). Nevertheless, the food environment in Latin America is shifting from healthier products to unhealthy food options, specifically due to the expansion of long-chain supermarkets, convenience stores, and fast-food restaurants (12, 15).

Since the creation of the North American Free Trade Agreement (NAFTA) in 1990, the availability of convenience stores that offer ultra-processed food, has increased in Mexico (16). From 2010 to 2018, the availability of convenience stores in urban environments grew by 142%, whereas the proportion of small grocery stores increased from 2 to 24% (17). Nonetheless, small grocery stores continue to be the most predominant type of traditional retailer in Mexico, where most of the impulsive and unplanned purchases are made (15).

The density of convenience and small grocery stores could contribute to diet quality. Evidence of the association between the density of this type of food stores and diet quality in Mexico is scarce. Most of the food environment evidence has focused on the availability of unhealthy food around schools (18, 19). Others described the increasing trends of stores offering ultra-processed food and their association with health outcomes in specific settings and populations (12, 18, 20, 21). Notwithstanding, little is known about the association between the density of these establishments and the consumption of certain types of food, as well as the whole diet. Thus, the aim of this article was to assess the association between the density of convenience and small grocery stores and diet quality in adults in Mexico City.

## Materials and methods

### Study design and data collection

We used data from the Mexico City Representative Diabetes Survey 2015 (MCRDS, 2015). This survey has a probabilistic multistage stratified cluster sampling design. Participants were selected through cluster sampling, using basic geostatistical areas (AGEB in Spanish acronym) as the primary sampling unit. From each AGEB, systematic sampling was conducted to six houses within six blocks. Two adults aged 20–69 years were systematically selected in each house. Trained personnel conducted face-to-face interviews using validated questionnaires between May and June 2015. The response rate for the original study was 71.4%. The MCRDS 2015 collected information on demographics, lifestyles, diet habits, and chronic diseases of 1,313 adults from Mexico City (22). We excluded adults with invalid or incomplete information on diet and those with potentially implausible intakes by food group and total energy > 3 S.D. (*n* = 299). Hence, the final analyses included 1,023 individuals.

### Variable definition

#### Mexican alternate healthy eating index

Diet data came from a validated semi-quantitative food frequency questionnaire (SFFQ). The SFFQ contained 140 foods that asked about participants' dietary intake over the previous seven days. This was administered by trained personnel using standardized data collection and entry procedures. Participants were asked to report the frequency of consumption of a standard portion of each food in the last 7 days and the times per day (one to six) (23). We estimated the diet quality for each participant by using the Mexican Alternate Healthy Eating Index (MxAHEI) (24). Details of the MxAHEI have been described elsewhere. Briefly, the MxAHEI includes 12 components: (1) vegetables, (2) whole fruit, (3) whole-grain cereals, (4) legumes, (5) seafood, poultry, or eggs, (6) polyunsaturated fat excluding long-chain fatty acids (EPA-eicosapentaenoic acid and DHA-docosahexaenoic acid), (7) long-chain fatty acids, (8) sugar-sweetened beverages, (9) red and processed meat, (10) sodium, (11) trans-fat, and (12) alcohol. Scores for each component ranged from 0 to 9. Zero “0” means that the individuals do not adhere to a recommended diet, whereas “9” represents those that fully adhere to a recommended diet. Solely, “5” for legumes and nuts refers to those that adhere to a recommended diet. Individuals that with a lower score, had a higher consumption of sugar-sweetened, red and processed meat, sodium, trans fat, and alcohol. Given the skewed distribution of the MxAHEI scores (total and by component), these were divided by the median into two categories: (1) high and (2) low consumption (24).

#### Density of grocery and convenience stores

We obtained the information on food retail in Mexico City from the 2015 National Statistical Directory of Economic Units (DENUE for its Spanish acronym). The information was collected by the National Institute of Geography and Statistics (INEGI for its Spanish acronym), based on the economic census conducted in 2014 (25). DENUE classifies economic units based on the North American Industrial Classification System (NAICS). Based on NAICS, we disaggregated “mini supermarkets” categories (code 462112) into small grocery stores (code 461110) and chain convenience stores (26). Spirits and beer outlets were excluded. We focus our analysis on these two types of food outlets because there are located nearby people's homes and offer ready-to-eat products (12, 17, 20, 21). We further considered for the analysis the presence of fruits and vegetable stores (fruterías y verdulerías), animal-based products stores (carnicerías, pollerías, marisquerías y pescaderías), supermarket, mini supermarkets stores, non-alcoholic beverage stores, bakeries (panaderías), corn tortilla stores (tortillería), sweets and confectionery stores (dulcerías) and ice cream parlors (heladerías) (Supplementary Table 1).

In order to verify if establishments were classified correctly, we manually reviewed the food outlet's name for DENUE food retail trade categories database. In cases where the food retail name or relevant information was not provided, we used Google maps street view to verify and insert the correct type of store.

#### Geocodification of individuals and food store

We used the Geographic Information System Software (QGIS) v3.10 to geocode the participants' homes and types of food outlets (27). Euclidean buffer of 500 meters was built around each participant's home (28). This buffer has been used in previous studies; in addition, this represents the walkability distance from home to any other place and is useful for cities with high connectivity, such as Mexico City (28–33). The density of small grocery, convenience stores, and other types of stores was defined as the total number of establishments within the buffer divided by the mean population of the census tract within the buffer zone (28–31). Then, the density of stores was standardized by dividing 1,000 inhabitants. In addition, based on previous studies, the density of stores by population was stratified into tertiles (18, 28, 29, 31, 32). Further information related to the density of stores per 1,000 inhabitants is described in Supplementary Table 2.

#### Covariates

Covariates included: sex, age, educational attainment, employment status, car possession, socioeconomic status, urban marginalization degree, and density of other stores. Age was classified into three categories (20 to 39 years, 40 to 59 years, and 60 years or more). We defined educational attainment as elementary school or less, middle school, and high school or more. Employment status was categorized into three groups: unemployed, 48 working or less per week, and working more than 48 per week (34). Car possession was dichotomized as yes or no. We constructed a socioeconomic status (SES) index by combining eight variables that assessed household characteristics, goods, and available services, including construction materials of the floor, ceiling, and wall; household goods (stove, microwave, washing machine, refrigerator, and boiler); and electrical goods (television, computer, radio, and telephone) (35). The index was divided into tertiles and used as a proxy for low, medium, and high SES. Urban marginalization degree was constructed based on the marginalization index calculated by National Population Council (CONAPO for its Spanish acronym), 2010. The index was based on variables aggregated at the municipal level that indicate the level of education (illiterate population or population without complete primary education aged 15 or over), the level of access to public services and housing conditions (without drainage, electricity, piped water, sanitary service, with overcrowding and earth floor), working and economic conditions (subsist with income up to 2 minimum wages) prevailing within each buffer zone determined for each household. This index was stratified into four levels: very low, low, medium, and high. Finally, the density of fruits and vegetable stores, animal-based products stores, supermarkets, mini supermarkets, non-alcoholic beverages stores, bakeries, corn tortilla stores, and ice cream parlors was split, at the median into categories of low density and high density (Supplementary Table 2).

### Statistical analyses

We first described the sociodemographic and lifestyle characteristics of the study sample. Second, we used Pearson's chi-squared test to examine the bivariate relationship of the density of small grocery and convenience stores and sociodemographic variables with the MxAHEI. Then we performed multivariable Poisson models to test the association of small grocery and convenience store densities with MxAHEI (overall and by component). The models were adjusted for sex, age, educational attainment, employment status, car possession, socioeconomic status, urban marginalization degree, and density of other types of stores. We considered the MxAHEI as the outcome variable using the high score as the reference category. We used the variance inflation factor (VIF) to check for multicollinearity. VIF values ranged from 1.22 to 3.26 suggesting no linear relationship among the predictors (36). The significance level was established at alpha 0.05. However, as recommended, we also described and discussed results that, even though not statistically significant, provide a broader picture of the findings (37). Statistical analyses were performed using Stata 14 (Stata corp LLC, College Station, TX, USA) (38). We used survey commands to account for survey design and weighting to generate nationally representative results.

## Results

A total sample of 1,023 adults was analyzed from the MCDRS 2015. As shown in Table 1, 63.2% are women, 43.4% are adults aged 40 to 59 years, and 41.7% had a higher level of schooling. More than half of adults worked 48 h or more per week (58.6%), and less than one-third owned cars (25.9%). The 31.8% of adults were classified with a high socioeconomic level, and 91.3% resided in areas with a very low to medium degree of marginalization.

**Table 1:** Sociodemographic characteristics of study participants.

**Characteristics**		***n* (%)**
Sex		
	Women	646 (63.2)
	Men	377 (36.8)
Age group		
	20–39	386 (37.7)
	40–59	444 (43.4)
	60 +	193 (18.9)
Educational attainment		
	Elementary school or less	283 (27.7)
	Middle school	313 (30.6)
	High school or more	427 (41.7)
Employment status		
	Unemployed	424 (41.4)
	48 working or less per week	378 (37.0)
	Working more than 48 per week	221 (21.6)
Car possession		
	Yes	265 (25.9)
	No	758 (74.1)
Socioeconomic status		
	Low	294 (28.7)
	Medium	404 (39.5)
	High	325 (31.8)
Urban marginalization degree		
	Very low	133 (13.0)
	Low	278 (27.2)
	Medium	523 (51.1)
	High	133 (8.7)
Mexican alternate healthy eating index		
	Low (15.40–37.18)	511(50.0)
	High (37.19–69.01)	512 (50.0)

The Mexico City representative diabetes survey, 2015 (n = 1,023).

Table 2 and Supplementary Table 3 show the characteristics of the study sample by the total MxAHEI score categories. We observed that the distribution of the total MxAHEI score was not statistically different across the density categories of grocery and convenience stores (Table 2). Likewise, we did not find differences in the total MxAHEI across densities of supermarkets, mini supermarkets, fruit and vegetable stores, corn tortilla stores, bakeries, and ice cream parlors (Supplementary Table 3). However, a higher number of adults with a high total MxAHEI score lived in buffers with a high density of fruit and vegetable stores. Otherwise, the total MxAHEI score distribution was statistically different across the density of animal-based products stores, sweets and confectionery stores, and non-alcoholic beverages stores. We found that more adults living in areas with a higher density of these stores had a lower total MxAHEI score (Supplementary Table 3).

**Table 2:** Mexican Alternate Healthy Eating Index scores by participants' characteristics.

**Characteristics**		**Mexican Alternate Healthy Eating Index** ^**a**^		
		**Low (15.40–37.18)**	**High (37.19–69.01)**	***p*-value**
		**% (CI 95%)**	**% (CI 95%)**	
Overall		52.7 (48.6,56.9)	47.2 (43.1,51.4)	
Density small grocery store ^b^
	Low (0.0–0.000092)	44.4 (37.3,51.8)	55.6 (48.2,62.7)	0.100
	Medium (0.000092–0.000236)	53.4 (46.5,60.2)	46.6 (39.8,53.5)	
	High (0.001490–0.000181)	56.0 (50.36,61.5)	44.0 (38.5,49.6)	
Density convenience store^b^
	Low (0–0)	48.7 (41.2,56.4)	51.2 (43.6, 58.9)	0.592
	Medium (0^.^000004–0.000016)	53.9 (45.5,62.0)	46.1 (38.0,54.5)	
	High (0.000016–0.000468)	54.3 (48.0,60.4)	45.7 (39.6,52.0)	
Sex				0.160
	Men	56.7 (48.7,64.2)	43.3 (35.7,51.3)	
	Women	49.6 (44.7,54.4)	50.4 (45.5,55.2)	
Age group				0.001
	20–39	58.9 (52.4,65.1)	41.1 (34.8,47.6)	
	40–59	47.1 (42.3,52.0)	52.9 (48.0,57.7)	
	60+	41.0 (33.2,49.1)	59.0 (50.8,66.7)	
Educational attainment				0.048
	Elementary school or less	46.0 (37.5,54.7)	54.0 (45.3,62.5)	
	Middle school	58.8 (52.1,65.2)	41.2 (34.8,47.8)	
	High school or more	52.0 (46.7,57.3)	48.0 (42.7,53.3)	
Employment status ^c^				0.072
	Unemployed	49.6 (43.2,56.1)	50.4 (43.9,56.8)	
	48 working or less per week	50.8 (44.2,57.3)	49.2 (42.7,55.8)	
	Working more than 48 per week	61.2 (52.6,69.2)	38.8 (30.8,47.3)	
Car possession				0.223
	Yes	49.8 (42.7,56.9)	50.2 (43.1,57.3)	
	No	54.1 (49.9,58.2)	45.9 (41.8,50.0)	
Socioeconomic status				0.197
	Low	46.4 (38.7,54.4)	53.5 (45.6,61.3)	
	Medium	55.8 (49.6,61.9)	44.2 (38.1,50.4)	
	High	53.2 (46.6,59.7)	46.7 (40.3,53.4)	
Urban marginalization				0.014
	Very low	41.9 (30.4,54.3)	58.1 (45.7,69.6)	
	Low	56.2 (49.5,62.6)	43.8 (37.4,50.5)	
	Medium	51.1 (45.8,56.3)	48.9 (43.7,54.2)	
	High	65.4 (56.6,73.3)	34.6 (26.7,43.4)	

^a^Mexican Alternate Healthy Eating Index classified as a binary variable by the median.

^b^Density stores: number of small grocery and convenience store per 1,000 habitants.

^c^Working hours defined by Mexican labor work.

A higher percentage of adults aged ≥60 had a higher total MxAHEI score than younger participants (<60 years). Also, a higher percentage of adults with elementary school or less had a higher MxAHEI score than those with higher educational attainment. Finally, a higher proportion of adults living in areas with a high degree of marginalization had a lower total MxAHEI score (Table 2).

Table 3 presents the association between the density of grocery stores and the total MxAHEI score adjusted for covariates. A lower prevalence of the total MxAHEI score was higher among adults residing in areas with a medium versus low density of grocery stores (PR = 1.18, 95% CI = 0.99, 1.38); however, this association was not statistically significant. Likewise, specifically by component, adults living in buffers with a medium and a high density of grocery stores had a higher prevalence of a low legumes score (PR = 1.09, 95%CI = 0.88, 1.35; PR = 1.20, 95%CI = 0.86, 1.68, respectively) and polyunsaturated fat score (PR = 1.08, 95%CI = 0.87, 1.34; PR = 1.06, 95%CI = 0.81, 1.06, respectively) compared to those living in areas with low density. Although these associations were not statistically significant.

**Table 3:** Association between the density of grocery stores with Mexican Alternate Healthy Eating Index overall and components^a^.

**MxAHEI^b^**	**Density of small grocery stores per Euclidean buffer** ^**c**^						
**Components**	***Low***	***Medium***				***High***	
	**(0.0–0.000092)**	**(0.000092, 0.000237)**				**(0.001490, 0.000181)**	
	**PR**	**PR**	**CI 95%**	***p*** **-value**	**PR**	**CI 95%**	***p*** **-value**
**Total score**							
Low score	1.00	1.18	(0.99, 1.38)	0.070	1.08	(0.87, 1.34)	0.484
**Vegetables**							
Low score	1.00	1.00	(0.80, 1.27)	0.957	0.93	(0.62, 1.39)	0.722
**Whole fruits**							
Low score	1.00	1.09	(0.88, 1.35)	0.441	1.01	(0.78, 1.32)	0.920
**Whole-grain cereals**							
Low score	1.00	1.00	(0.90, 1.15)	0.986	0.96	(0.79, 1.16)	0.643
**Legumes**							
Low score	1.00	1.09	(0.88, 1.35)	0.416	1.20	(0.86, 1.68)	0.281
**Nuts**							
Low score	1.00	0.97	(0.82, 1.14)	0.708	0.81	(0.66, 1.01)	0.059
**Polyunsaturated fat**							
Low score	1.00	1.08	(0.87, 1.34)	0.483	1.06	(0.81, 1.40)	0.642
**Long-chain (n-3) fats (EPA+DHA)**							
Low score	1.00	0.93	(0.71, 1.22)	0.609	0.80	(0.60, 1.05)	0.105
**Sugar-sweetened beverages**							
Low score							
(High intake)	1.00	0.94	(0.84, 1.04)	0.236	0.94	(0.83, 1.05)	0.282
**Red and processed meat**							
Low score							
(High intake)	1.00	1.02	(0.96, 1.08)	0.517	1.02	(0.94, 1.11)	0.577
**Sodium**							
Low score							
(High intake)	1.00	1.02	(0.95, 1.09)	0.593	1.05	(0.95, 1.16)	0.323
**Trans fat**							
Low score							
(High intake)	1.00	0.98	(0.94, 1.03)	0.487	0.96	(0.91, 1.01)	0.105
**Alcohol**							
Low score							
(High intake)	1.00	0.95	(0.86, 1.04)	0.230	0.91	(0.81, 1.01)	0.089

^a^Models were adjusted for sex, age, socioeconomic status, educational attainment, working hours per week, car possession, urban marginalization, and the density of fruits and vegetable stores, animal-based products stores, supermarkets, mini supermarkets, non-alcoholic beverages stores, bakeries, corn tortilla stores, and ice cream parlors.

^b^Mexican Alternate Healthy Eating Index (MxAHEI) scores classified as binary variables by the median. The reference category is high score (high intake for healthy components and low intake for unhealthy components).

^c^Density of grocery stores per 1,000 inhabitants.

^d^PR, Prevalence Ratio.

Table 4 shows the association of the density of convenience stores with the total MxAHEI score adjusted for covariates. There was no statistically significant association between convenience stores and the overall MxAHEI score. However, the prevalence of a low total MxAHEI score was higher among adults living in buffers with a medium and high vs. low density of convenience stores (PR = 1.15, 95%CI = 0.92,1.43; PR = 1.14, 95%CI = 0.93,1.40, respectively). Moreover, we found a statistically significant association between adults living in areas with a medium and higher density of convenience stores and a lower prevalence of a low polyunsaturated fat score (PR = 0.78, 95%CI = 0.69, 0.94; PR = 0.76, 95%CI = 0.60, 0.95, respectively). Regarding other components, we did not find a statistically significant associations; however, a high prevalence of a low vegetable score was observed among adults residing in areas with medium and high vs. low density of convenience stores (PR = 1.19, 95% CI = 0.98, 1.45: PR = 1.13, 95%CI 0.92, 1.39, respectively). Furthermore, adults living in areas with a medium and high density of convenience stores had a higher prevalence of a low legumes score (PR = 1.17, 95%CI = 0.94, 1.46; PR = 1.18, 95%CI = 0.95, 1.47, respectively) and long-chain (n-3) fats (EDA+DHA) score (PR = 1.04, 95%CI = 0.79, 1.37; PR = 1.14, 95%CI = 0.92, 1.42, respectively) compared to those living in areas with low density. Nevertheless, adults living in areas with medium and high density had a higher intake (low score) of processed meat (PR = 1.02, 95%CI = 0.95, 1.09; PR = 1.06, 95%CI = 0.98, 1.14, respectively) compared to adults living in areas with low density.

**Table 4:** Association between the density of convenience stores with Mexican Alternate Healthy Eating Index overall and components^a^.

**MxAHEI ^b^**	**Density of convenience stores per Euclidean buffer** ^**c**^						
**Components**	***Low***	***Medium***				***High***	
	**(0, 0)**	**(0.000004, 0.000016)**				**(0.000016, 0.000468)**	
	**PR**	**PR**	**CI 95%**	***p*** **-value**	**PR**	**CI 95%**	***p*** **-value**
**Total score**							
Low score	1.00	1.15	(0.92, 1.43)	0.207	1.14	(0.93, 1.40)	0.185
**Vegetables**							
Low score	1.00	1.19	(0.98, 1.45)	0.083	1.13	(0.92, 1.39)	0.232
**Whole fruits**							
Low score	1.00	0.95	(0.76, 1.19)	0.636	0.90	(0.74, 1.10)	0.294
**Whole-grain cereals**							
Low score	1.00	0.97	(0.87, 1.10)	0.676	0.94	(0.84, 1.06)	0.344
**Legumes**							
Low score	1.00	1.17	(0.94, 1.46)	0.159	1.18	(0.95, 1.47)	0.121
**Nuts**							
Low score	1.00	0.99	(0.89, 1.10)	0.868	0.94	(0.82, 1.07)	0.342
**Polyunsaturated fat**							
Low score	1.00	0.78	(0.69, 0.94)	**0.012** ^**e**^	0.76	(0.60, 0.95)	**0.019** ^**e**^
**Long-chain (n-3) fats (EDA+DHA)**							
Low score	1.00	1.04	(0.79, 1.37)	0.760	1.14	(0.92, 1.42)	0.225
**Sugar-sweetened beverages**							
Low score							
(High intake)	1.00	1.00	(0.91, 1.11)	0.910	0.99	(0.90, 1.08)	0.802
**Red and processed meat**							
Low score							
(High intake)	1.00	1.02	(0.95, 1.09)	0.580	1.06	(0.98, 1.14)	0.145
**Sodium**							
Low score							
(High intake)	1.00	0.99	(0.94, 1.04)	0.807	1.00	(0.94, 1.07)	0.868
**Trans fat**							
Low score							
(High intake)	1.00	0.98	(0.93, 1.03)	0.369	0.97	(0.92, 1.02)	0.227
**Alcohol**							
Low score							
(High intake)	1.00	0.99	(0.94, 1.06)	0.961	0.99	(0.92, 1.06)	0.774

^a^Models were adjusted for sex, age, socioeconomic status, educational attainment, working hours per week, car possession, urban marginalization, and the density of fruits and vegetable stores, animal-based products stores, supermarkets, mini supermarkets, non-alcoholic beverages stores, bakeries, corn tortilla stores, and ice cream parlors.

^b^Mexican Alternate Healthy Eating Index (MxAHEI) scores classified as binary variables by the median. The reference category is high score (high intake for healthy components and low intake for unhealthy components).

^c^Density of grocery stores per 1,000 inhabitants.

^d^PR, Prevalence Ratio.

^e^Statistically significant p < 0.05.

## Discussion

To our knowledge, this is one of the first studies that analyzed the association of the density of small grocery and convenience stores with the diet quality of adults living in Mexico City. We found that the small grocery stores were not statistically associated with quality diet, overall or by component. However, we did observe a lower diet quality score in buffers with medium vs. low density of small grocery stores. Specifically, a higher percentage of adults living in areas with medium and high density of small grocery stores had lower scores for legumes and polyunsaturated fat. Regarding the convenience stores, although not statistically significant, we found that a medium and high density of these stores was associated with a lower score in the overall diet quality. Also, a higher number of adults living in areas with a medium and high density of convenience stores had lower scores for vegetables, legumes, and long-chain (n-3) fats. However, we observe that a high and medium density of convenience stores was statistically associated with higher polyunsaturated fat intake.

### Grocery stores and diet quality

Our findings of an inverse association not statistically significant between a higher density of small grocery stores and diet quality score can be explained by several reasons. First, the results seem to be consistent with what these establishments offer. Bridle-Fitzpatrick et al., studied the food environment in a city in a northern state of Mexico (Mazatlán, Sinaloa); they found that small grocery stores sold a small selection of fresh fruit and vegetables and large quantities of sugar-sweetened beverages (SSBs) and packaged snacks (20). Second, although the number of convenience stores has increased, the small grocery stores (*tiendas de abarrotes*) continue to be the most predominant food retailer in Mexico; there are 67 small retail stores per chain convenience store (12). Small grocery stores have great cultural importance, affordable prices, are located nearby homes, and offer credit to the customers (15), which might promote the intake of unhealthy and energy-dense foods at a lower cost. Moreover, it has been documented that small grocery stores account for an important proportion of the sales of transnational food companies in Mexico, including SSBs, baked goods, snacks, beer, and dairy (39).

Although not statistically significant, we observed an inverse association between small grocery store density and legumes intake. Even though small grocery stores offer dry and canned legumes, we do not exclude the possibility that people purchase their legumes from other stores like supermarkets (40). However, we do not have information on purchases of legumes by type of food store. Moreover, the estimations could be a reflection of the low intake of legumes in Mexico. Aburto et al. (41), found that legumes had the lowest energy contribution (3.8%) of the total energy intake among the Mexican population, especially in urban localities (42).

We did not observe an association between the density of small grocery stores and other diet components (vegetables, whole fruits, whole grains, seeds, red and processed meats, long-chain (n-3) fats (EPA+DHA), SSBs, trans-fat, alcohol). One possible explanation of this finding is that these foods are found in fresh food stores in Mexico (e.g., fruit and vegetable stores, butchers, poultry shops, fishmongers, and other specialized shops) (17, 20), and their purchases are likely independent of what is acquired in small grocery stores.

### Convenience stores and diet quality

Although not statistically significant, we found an inverse association between convenience stores and the overall diet quality. In the same line, Lind et al., found that higher availability of convenience stores within 500 meters network buffers was associated with an unhealthy diet among 16 years and older Danish (32). Also, a longitudinal study in young adults in the United States found an association between a higher density of convenience stores and a low score quality of the diet for participants living within a 3-kilometer distance along the street network (7). One potential explanation for our not statistically significant results could be that the number of convenience stores in Mexico is still small relative to small grocery stores (12, 21). However, the relative proportion of convenience stores continues to rise (12, 15, 17, 21). Therefore, monitoring the association between convenience stores and diet intake will be needed. Also, the density of convenience stores is more likely to be related to specific food components than the overall diet.

Specifically, we found a not statistically significant inverse association between convenience store density and the vegetable component score. This finding is consistent with the study by Zhang et al. (43), who observed that a higher density of convenience stores was associated with an increased likelihood of infrequent vegetable consumption in the Chinese population (41). Another study performed in an urban multiethnic population in the United States found that the presence of a convenience store in the neighborhood was negatively associated with vegetable intake among Latinos (7). Although convenience stores represent a small proportion of the total stores (1.3% in 2016), it is the fastest-growing store type in Mexico (12). The availability of convenience stores could negatively influence the consumption of healthy food products because convenience stores typically carry fewer whole grains, legumes, fruit, and vegetables than other retail food outlets. A recent study in Mexico showed that the density of convenience stores was associated with higher purchases of ultra-processed food and SSBs, probably linked to their low prices and large availability in these stores (15).

We also found that a high and medium density of convenience stores was statistically associated with a higher intake of polyunsaturated fat, excluding long-chain fat. This finding can be explained by the fact that vegetable oils, highly accessible in convenience stores, are a major source of no-long-chain fat in Mexico (43). Although polyunsaturated fats can have a protective role against cardiovascular disease, this effect could be detrimental when used to fried the food (44, 45). Future analysis will be needed to determine the type of polyunsaturated fats purchased by the retail food store and how they are consumed.

### Strengths and limitations

Our study has several limitations. First, this was a cross-sectional study; therefore, it is not possible to determine the causal relationship between grocery and convenience store density and diet quality. However, the findings highlight the potential of the food environment to influence the diet of adults in Mexico City. Future longitudinal studies will be needed to confirm our results. Second, the data analyzed are not recent. However, the association between the density of food outlets and diet quality is not expected to change significantly over a short time. Third, we do not rule out the possibility of selection bias since the missingness in diet information might be related to the diet itself. Fourth, our findings could be only extrapolated to areas with populations with a socioeconomic status distribution similar to our study sample, which was different from those observed in the excluded sample (Supplementary Table 4). Moreover, although the original study might be representative of adults from Mexico City, this might not be true by geographic region. Given the nature of our analyses, it is likely that more individuals were clustered in some buffers than others. Therefore, our no statistically significant results might be partially explained by the low variability in the exposure and outcome due to the geographic distribution of our study sample. The dichotomization of the outcome variable can also explain the low variability in the MxAHEI scores across buffers and, therefore, the non-significant results. Nevertheless, we considered the results informative given the directions of the estimations. Fifth, we estimated store density to quantify the availability of food stores by using geographic information methods only (46). However, we did not conduct any store audit or checklist to assess the food availability in the food outlets of interest in more detail (47). Sixth, we did not consider other types of food stores, such as informal or mobile food outlets (as the DENUE data do not include information about informal food vendors), which could impact the diet, as shown in other studies (17, 18). Moreover, as mentioned before, we focused on small grocery and convenience stores because of their proximity to people's homes and their supply of products with immediate access (12, 17, 20). Seventh, although Euclidean buffer zones have limitations concerning street network buffers, both metrics have similar correlation results in terms of the level of pseudo individual density (at the same distance), and the correlation increased so when accounting for larger neighborhoods (400 m r_s_ = 0.667 and 800 m r_s_ = 0.667 *p* < 0.001) (28). Eighth, we focused on the food environment around homes in a buffer of 500 meters; however, likely individuals do not shop (or not always) at small grocery or convenience stores near home. Nineth, one of the main limitations of the SFFQ is it might underestimate the food intake; however, it is not a limitation in our study since the MxAHEI considers the total energy intake. Finally, we recognized that the MxAHEI scores calculated might not reflect the variability in dietary intake by seasonality since the survey was conducted between May and June. Future studies considering food information from different seasons would provide further insights into the potential association between the food environment and diet quality. For future studies, it is important to consider combining multiple evaluation techniques, including individual factors and social contexts of Mexican populations, for example, promotion, price, availability of homemade food/beverages, and acceptability, which have been linked with unhealthy diets (19). Therefore, counting more of these variables would help better understand the mechanisms for which the food environment potentially affects the food intake.

This study's strength includes using an economic census database to estimate the density of convenience stores rather than self-report of stores. Also, this is one of the first studies that explore the association between the density of two types of food retails (small grocery and convenience stores) and the diet quality among Mexican adults. This study contributes to the literature on the community food environment.

## Conclusion

We observed an association not statistically significant between a higher density of small grocery stores and convenience stores and lower overall diet quality among adults in Mexico City. Despite the limitations, this study adds new insights to understanding the role of the grocery and convenience stores in adopting a healthy diet, regardless of individual characteristics. Nevertheless, more evidence is required to narrow the knowledge gap on the underlying causes of unhealthy dietary patterns by making the food environment visible as a risk factor.

## Data availability statement

The original contributions presented in the study are included in the article/Supplementary material, further inquiries can be directed to the corresponding author.

## Author contributions

AIR-G, NL-O, and CM: conceptualization. AIR-G and NL-O: diet quality analysis. AIR-G, CH-A, and AGO-A density stores analysis. AIR-G, NL-O, and AGO-A: statistics analysis. AIR-G, NL-O, CM, CH-A, and AGO-A: investigation and methodology. NL-O, CM, CH-A, and AGO-A: supervision, validation, writing, review, and editing. AIR-G: writing—original draft preparation. SB conceived, designed, and executed the original study. All authors approved the submitted version.

## Funding

AIR-G received a master's degree fellowship from the National Council of Science and Technology of Mexico.

## Conflict of interest

The authors declare that the research was conducted in the absence of any commercial or financial relationships that could be construed as a potential conflict of interest.

## Publisher's note

All claims expressed in this article are solely those of the authors and do not necessarily represent those of their affiliated organizations, or those of the publisher, the editors and the reviewers. Any product that may be evaluated in this article, or claim that may be made by its manufacturer, is not guaranteed or endorsed by the publisher.
