# Supplementary material for: Association between density of convenience and small grocery stores with diet quality in adults living in Mexico City: A cross sectional study
Source: Front Public Health. 2022 Aug 5;10:857754. doi: 10.3389/fpubh.2022.857754 (PMC9389155; doi:10.3389/fpubh.2022.857754)
Supplement: Supplementary file 1 [file Data_Sheet_1.docx]

Supplementary Material

| **Supplementary Table 1. North American Industrial Classification (NAICS) codes used to define food store types in the present study.** | | |
| --- | --- | --- |
| Food store type | NAICS code^26^ | Characteristic^12,21^ |
| Small grocery store | 461110 | Type of traditional food outlets that sell various of products including staple foods, a small selection of fruit and vegetables, as well as ultra-processed foods. |
| Chain convenience stores | Disaggregated from code 462112  Searched by name because NAICS does not identified them as a distinct store format. | Open≥18 h a day for 365 days a year, and sell mainly processed and ultra-processed food products and beverages (e.g., OXXO, 7-Eleven, Circle K). |
| Supermarkets | 462111 | Grocery stores and wholesale outlets that sell both healthy and unhealthy food options (e.g., Walmart, Superama, Bodega Aurrera, Costco). |
| Mini supermarkets | 462112 | Outlets that sell food, beverages, and other household products. These establishments are smaller than supermarkets but can be bigger than small grocery shops. |
| Fruit and vegetable stores | 461130 | Permanent establishments that exclusively sell fruits and vegetables. |
| Animal-based products stores | 461121  461122  461123  461150 | Small food outlet that specializes in selling poultry meat and offal, raw or semi-cooked red meat and offal, fish and seafood (fresh, dried, salted and frozen). Milk and other dairy products, cooked meats. |
| Bakeries | 311812  Disaggregated from 461190 | Bread, traditional sweet bread, cakes, pasties. |
| Corn tortilla stores | 311830  Disaggregated from 461190 | Food outlets dedicated to the production and sale of corn tortillas. Some of these outlets also sell wheat tortillas. |
| Sweets and confectionery stores | 461160 | Outlets that sell sweets, chocolates, regional sweets, as well as raw materials used in the production of confectionary products. |
| Ice cream parlors | 461170 | Outlets that sell ice cream and popsicles. |
| Non-alcoholic beverages | 461213 | Outlets that sell mainly non-alcoholic packaged beverages, such as soft drinks, juices and nectars, sports drinks, and energy drinks. |

| **Supplementary Table 2. Density of food stores.** | | |
| --- | --- | --- |
| Food store type ^a^ | Density of food store per 1000 inhabitants | |
|  | Low | High |
| Supermarkets | 0-0 | 0.000002 - 0.000021 |
| Mini supermarkets | 0-0.000007 | 0.000007-0.000055 |
| Fruit and vegetable stores | 0 - 0.000074 | 0.000074 - 0.011978 |
| Animal-based products stores | 0 - 0.000074 | 0.000074 - 0.011978 |
| Bakeries | 0-0.000043 | 0.000043-0.000149 |
| Corn tortilla stores | 0-0.000060 | 0.000061-0.0002463 |
| Sweets and confectionery stores | 0-0.000047 | 0.000047-0.003356 |
| Ice cream parlors | 0-0.000014 | 0.000014-0.000074 |
| Non-alcoholic beverages stores | 0-0.000009 | 0.000009-0.000573 |
| ^a^ Food stores were classified as a binary variable by the median. | | |

| **Supplementary Table 3. Mexican Alternate Healthy Eating Index scores by the density of food stores.** | | | | |
| --- | --- | --- | --- | --- |
| Density of food stores per 1000 inhabitants | | Mexican Alternate Healthy Eating Index ^a^ | | |
|  |  | Low  (15.40 – 37.18) | High  (37.19- 69.01) |  |
|  |  | % (CI 95%) | % (CI 95%) | p value |
| Supermarkets |  |  |  | 0.484 |
|  | Low  (0 - 0)  High  (0.000002 - 0.000021) | 51.9 (47.0,56.8)  54.7 (48.0,61.2) | 48.1 (43.2,53.0)  45.3 (38.8,52.0) |  |
| Mini supermarkets |  |  |  | 0.651 |
|  | Low  (0-0.000007) | 51.8 (46.2,57.3) | 48.2(42.7,53.8) |  |
|  | High  (0.000007-0.000055) | 53.8 (47.3,60.2) | 46.2 (39.8,52.7) |  |
| Fruit and vegetable stores |  |  |  | 0.053 |
|  | Low  (0 - 0.000074)  High  (0.000074 - 0.011978) | 49.4 (43.2,55.5)  56.5 (52.2,60.7) | 50.6 (44.4,56.7)  43.5 (39.3,47.8) |  |
| Animal-based products stores |  |  |  | 0.045 |
|  | Low  (0-0.000043) | 49.1 (42.5,55.7) | 50.9 (44.3,57.4) |  |
|  | High  (0.000043-0.000149) | 56.9 (52.7,61.0) | 43.1 (38.9,47.3) |  |
| Bakeries |  |  |  | 0.316 |
|  | Low  (0-0.000043) | 47.4 (35.3,59.8) | 52.6 (40.2,64.7) |  |
|  | High  (0.000043-0.000149) | 51.1 (39.7,62.4) | 48.9 (37.6,60.3) |  |
| Corn tortilla stores |  |  |  | 0.052 |
|  | Low  (0-0.000060) | 49.3 (43.4,55.3) | 50.7 (44.7,56.6) |  |
|  | High  (0.000061-0.0002463) | 56.8 (51.8,61.7) | 43.2 (38.3,48.2) |  |
| Ice cream parlors |  |  |  | 0.106 |
|  | Low  (0-0.000014) | 49.7 (43.8,55.7) | 50.3 (44.3,56.2) |  |
|  | High  (0.000014-0.000074) | 55.9 (50.8,60.8) | 44.1 (39.1,49.2) |  |
| Sweets and confectionery stores |  |  |  | 0.006 |
|  | Low  (0-0.000047) | 48.1 (42.5,53.7) | 51.9 (46.3,57.4) |  |
|  | High  (0.000047-0.003356) | 58.2 (53.3,62.9) | 41.8 (37.1,46.7) |  |
| Non-alcoholic beverages stores |  |  |  | 0.020 |
|  | Low  (0-0.000009) | 48.2 (41.9,54.7) | 51.8 (45.3,58.1) |  |
|  | High  (0.000009-0.000573) | 57.8 (52.8,62.7) | 42.2 (37.3,47.2) |  |
| ^a^ Mexican Alternate Healthy Eating Index classified as a binary variable by the median.  ^b^ p-value<0.05 | | | | |

| **Supplementary Table 4. The distribution of covariables between the included and excluded sample of study participants.** | | | | |
| --- | --- | --- | --- | --- |
| Characteristics | | Included  (n=1023) | | Excluded (n= 290) |
| Sex |  | |  |  |
|  | Women | | 646 (63.2) | 179 (61.7) |
|  | Men | | 377 (36.8) | 111 (38.3) |
| Age group |  | |  |  |
|  | 20 - 39 | | 386 (37.7) | 111 (38.8) |
|  | 40 - 59 | | 444 (43.4) | 126 (43.45) |
|  | 60 + | | 193 (18.9) | 53 (18.28) |
| Educational Attainment |  | |  |  |
|  | Elementary school or less | | 283 (27.7) | 87 (30) |
|  | Middle school | | 313 (30.6) | 74 (25.5) |
|  | High school or more | | 427 (41.7) | 129 (44.5) |
| Socioeconomic Status |  | |  |  |
|  | Low | | 294 (28.7) | 114 (39.3) |
|  | Medium | | 404 (39.5) | 85 (29.3) |
|  | High | | 325 (31.8) | 91 (31.4) |
